# Validity of information on atopic disease and other illness in young children reported by parents in a prospective birth cohort study

**DOI:** 10.1186/1471-2288-12-160

**Published:** 2012-10-22

**Authors:** Nadja Hawwa Vissing, Signe Marie Jensen, Hans Bisgaard

**Affiliations:** 1 Copenhagen Prospective Studies on Asthma in Childhood, The Danish Pediatric Asthma Center, Health Sciences, University of Copenhagen, Copenhagen University Hospital, Gentofte, Ledreborg Alle 34 2900 Hellerup, Copenhagen, Denmark

**Keywords:** Validation studies, Cohort Studies, Child, Asthma, Atopic dermatitis, Diagnosis, Interviews, Recall bias, Infections

## Abstract

**Background:**

The longitudinal birth cohort study is the preferred design for studies of childhood health, particularly atopic disease. Still, prospective data collection depends on recollection of the medical history since the previous visit representing a potential recall-bias. We aimed to ascertain the quality of information on atopic disease and other health symptoms reported by parental interview in a closely monitored birth cohort study. Possible bias from symptom severity and socioeconomics were sought.

**Methods:**

Copenhagen study on Asthma in Childhood (COPSAC) is a clinical birth cohort study of 411 children born of asthmatic mothers from 1999 to 2001. Child health is monitored at six-monthly visits with particular emphasis on atopic symptoms and infections. Data from the first three study years on 260 children was compared with records from their family practitioner as an external reference.

**Results:**

A total of 6134 medical events were reported at the COPSAC interviews. Additional 586 medical events were recorded by family practitioners but not reported at the interview. There were no missed events related to asthma, eczema or allergy. Respiratory, infectious and skin related symptoms showed completeness above 90%, other diseases showed lower completeness around 77%. There was no meaningful influence from concurrent asthma or socioeconomics.

**Conclusions:**

The COPSAC study exhibited full sensitivity to the main study objectives, atopic disease, and high sensitivity to respiratory, infectious and skin related illness. Our findings support the validity of parental interviews in longitudinal cohort studies investigating atopic disease and illness in childhood.

## Background

The longitudinal birth cohort study is the preferred design for studies of the origins of chronic diseases in childhood such as asthma [1,2], partly because recall bias is minimized. The prospective clinical cohort study with doctor’s interview and examination at regular visits to the clinic is expected to have the highest completeness capturing medical and exposure history. Recall bias is reduced with a high frequency of visits to the clinic, but information still depends on recollection of the medical history since the previous visit. Particularly short-term symptoms such as common childhood infections may be influenced by recall bias [3,4]. Diary data is considered a valid source and provides strong support to the history taking at the clinic. But such data collection also depends on a high level of compliance and the risk of data being influenced by socioeconomics of the subjects [4,5].

The validity of information obtained in prospective clinical cohort studies have been sparsely investigated, in part because this requires a comparison with an external standard, which is rarely available [6,7].

The Copenhagen Prospective Study on Asthma in Childhood (COPSAC) is an ongoing clinical, prospective, longitudinal birth-cohort study of 411 infants born to mothers with asthma designed to intensively investigate the development of atopic diseases in high-risk children, with a dedicated research clinic providing the families ready access to clinical evaluation and treatment with scheduled and acute clinical visits. Any atopic or respiratory illness is seen and treated by the research doctors. Precise case definition of atopic disease is the hallmark of the COPSAC cohort study. Nevertheless, as dedicated as the research unit can be, it still requires a high level of compliance from study participants. Children that are diagnosed and treated by other physicians without our knowledge would potentially compromise the quality of our data.

We aimed to ascertain the completeness of our data on atopic diseases during the first three years of life in an ongoing prospective clinical birth cohort study. Secondly we aimed to investigate potential underreporting of the history of symptoms, diagnoses and other health care resource utilization reported by interview at six-monthly visits at our clinical research unit. Information collected by the family’s General Practitioner was used as external reference for such missing data. As a part of this analysis we tried to look at bias in parent-reporting related to the child’s asthma status and socioeconomics of the family.

## Methods

### Study cohort

COPSAC is a longitudinal birth cohort study designed to examine the relation between the genetics, environmental and lifestyle factors and development of asthma, eczema and allergic symptoms in early life. The cohort study design and characteristics have previously been described in detail [8].

Subjects participating in COPSAC were recruited between August 1998 and December 2001 among pregnant mothers with a history of asthma diagnosed by a doctor and requiring medication. 411 children of asthmatic mothers were included in a comprehensive program of clinical and objective assessments. At three years of age 350 children (85%) were still active in the cohort. Children attended the COPSAC clinic for planned visits at one month of age and every six months. Additionally they were seen for any acute airway and/or skin symptoms. At each six-monthly visit, the medical doctors at COPSAC interviewed the parents about any illnesses, symptoms and use of medication during the previous six months. The doctor assessed the reported illnesses, asking clarifying questions when needed, and classified them as ‘medical events’ by ICD10 codes. Medical events were entered online into the COPSAC database with a start and end date for each episode of illness. If a child was seen during a medical event, the end date was added at the following interview session.

All data were collected according to Good Clinical Practice data management and quality control procedures including external monitoring. The study is conducted in accordance with the Declaration of Helsinki and approved by the Copenhagen Ethics Committee (KF01-289/96) and the Danish Data Protection Agency (2008-41-1754). Written informed consent was obtained from both parents/legal guardians.

### Disease categories

In the present analysis we classified all diagnoses into the following five groups: 1. Atopic diseases (asthma, atopic dermatitis and allergic rhinitis), 2. Airway related illness (upper and lower airway infections), 3. Skin related illness (all diagnoses related to skin other than atopic dermatitis), 4. Childhood infections (infections with fever, without airway symptoms) and 5. Other illness. For group details see Table 1.

**Table 1:** Frequencies and completeness of diagnoses in groups

	**COPSAC (n)**	**Missed events**	**Total (N)**	**Completeness (n/N)**
**Atopic disease (Ever)**	**172**	**0**	**172**	**1.00**
Asthma	48	0	48	1.00 [0.94, 1.00]*
Atopic dermatitis	113	0	113	1.00 [0.97, 1.00]*
Allergic rhinitis	11	0	11	1.00 [0.76, 1.00]*
**Airway related disease**	**3104**	**154**	**3258**	0.95 [0.94, 0.96]**
Common cold	1690	61	1751	0.97
Otitis media	587	44	631	0.93
Pneumonia	264	11	275	0.96
Cough	208	23	231	0.90
Tonsilitis	130	10	140	0.93
Tubulation	106	1	107	0.99
Croup	85	4	89	0.96
RS-virus	33	0	33	1.00
Pertussis	1	0	1	1.00
**Skin related disease**	**878**	**49**	**927**	0.95 [0.93, 0.96]**
Dermatitis	438	13	451	0.97
Dry skin	166	0	166	1.00
Erythema	96	13	109	0.88
Nappy dermatitis	93	7	100	0.93
Impetigo	41	6	47	0.87
Urticaria	32	8	40	0.80
Herpes infection	12	2	14	0.86
**Childhood infections**	**969**	**88**	**1057**	0.92 [0.89, 0.94]**
Fever	684	81	765	0.89
Chicken pox	110	1	111	0.99
3-day fever	72	6	78	0.92
Influenza	63	0	63	1.00
Fever cramps	25	0	25	1.00
Erythema infectiosum	8	0	8	1.00
Scarlet fever	7	0	7	1.00
**Other illness**	**1010**	**295**	**1305**	0.77 [0.74, 0.80]**
Diarrhea and Vomiting	417	62	479	0.87
Conjunctivitis	252	114	366	0.69
Candidiasis	68	31	99	0.69
Constipation	28	10	38	0.74
Minor trauma	18	37	55	0.33
Adenectomy	14	0	14	1.00
Insect bite	11	5	16	0.69
Urinary Tract infection	10	1	11	0.91
Molluscs	9	2	11	0.82
Abscess	6	2	8	0.75
Mononucleosis	3	0	3	1.00
Septicaemia	3	0	3	1.00
Burn	2	6	8	0.25
Other, non-infectious events	169	25	194	0.87
**Total**	**6134**	**586**	**6719**	0.91 [0.90,0.92]**

* Exact one-sided lower 95% confidence interval.

** Two-sided 95% confidence interval.

### Atopic disease

#### Asthma

Since the study population comprised children below 3 years of age the diagnosis of asthma was based largely on symptoms, according to international guidelines [9]. Diagnosis of asthma was based on a predefined algorithm [9,10] focusing on persistent wheezy symptoms and subsequent response to treatment. Respiratory symptoms were recorded by the parents in daily diaries. The description of symptoms was supported by a book (written for parents, about early childhood wheeze) that was integrated with the diary cards. The COPSAC doctors reviewed the diary entries with the parents at the 6 monthly visits as well as during acute episodes of wheeze. A wheezy episode was defined on the diary card as 3 consecutive days of wheeze. Persistent wheeze was defined as five such episodes within 6 months or daily symptoms for 4 consecutive weeks leading to a 3-month course of inhaled corticosteroids (ICS). Acute severe asthmatic exacerbation also led to a 3-month course of ICS. Children responding to treatment and with a relapse when stopping treatment were diagnosed with asthma. Further treatment followed a strict algorithm previously described in details [11,12].

Atopic dermatitis was defined by the criteria of Hanifin and Rajka based on the presence of 3 of 4 major criteria and at least 3 of 23 minor signs as previously detailed [13].

Allergic rhinitis was diagnosed in children with seasonal symptoms and sensitization to relevant allergens measured by a specific IgE test [14]. Symptoms were defined as persistent troublesome sneezing or blocked or runny nose severely affecting the well-being of the child in periods without common cold, fever or flu [15].

Parents were requested to contact the COPSAC clinic if their child developed any kind of airway or skin related symptoms. If a child developed asthma, eczema or allergic rhinitis, it attended the clinic every three months for clinical evaluation and additionally at acute disease exacerbation. All medical treatment was handled by the COPSAC clinic.

In the present study atopic diseases were viewed as life-time diagnoses in the follow-up period, i.e. the child was diagnosed at any time point within the first 3 years of life.

### External standard

The GP occupies a central position in the Danish health care service and the GP records are considered a reliable external data source. Citizens are signed up with a GP of his own choice and are allowed to change only once a year. The GP is the patients’ primary contact with the health service and act as "gate keeper" to secondary care specialists. The GP keeps a record on every patient. A personal identification number is assigned to every Danish citizen linking all health care utilizations to the patient. If a patient seeks other medical health care, such as outpatient clinic, emergency room and hospital, a discharge summary is sent to the GP. Consequently no patients are treated for an illness without automatic notification of the GP, and any illness severe enough to cause medical attention, even by phone, is registered by the GP. Prescription rules in Denmark are strict and only very few drugs can be bought over the counter without doctor’s prescription, which excludes patients from use of any antibiotics or anti-asthmatics without prescription automatically recorded by the GP.

The family practitioner (GP) of every COPSAC child was identified in the national health registry. Written informed consent to retrieve information from other health care sources, including the GP, was obtained from both parents/legal guardians. The GP of children who completed three study years was requested by mail to send a copy of his record from the child’s first three years of life. When the records were returned they were reviewed by a trained senior medical student and compared with COPSAC database information. The same person reviewed all the records. When an event was captured by the GP but not by COPSAC, it was considered as a missed event and was registered in a separate data sheet by ICD10 code and dated. Sometimes dates of the same type of event differed between COPSAC and GP records. In such cases a time span of four weeks between the registered start dates was allowed.

### Socioeconomics

As a proxy for socioeconomic status information on household income, educational level and occupational status of the mother was obtained at the one-year-visit. Household income was classified into three groups: Income below average (<400.000 DKR), around average (400.000 – 600.000 DKR) and above average (>600.000 DKR) [8]. The highest level of completed education was divided into four categories: elementary school, college, medium and university education. Occupational status was described in four groups; professionals, non-professionals, unemployed and student; based on DISCO classification, a national statistic used by the Danish National Statistic Agency ( http://www.dst.dk).

### Statistical analysis

The total number of medical events for a child (N) was considered as the sum of events registered by COPSAC (n) plus the additional missed events found in the GP record. The completeness of COPSAC information was estimated as the number of medical events recorded by COPSAC (n) as a percentage of the total number of medical events (N).

Completeness was estimated using a GEE model with the logit link function taking into account the child variation. P-values corresponded to score tests. Confidence intervals of sensitivities were estimated on a logit scale, back-transformed and presented in brackets. Analyses were done using PROC GENMOD in SAS 9.1.

## Results

The first three years of longitudinal data collection was completed by 350 children. 327 (93%) GPs responded to the request of a copy of the child’s record. Of the returned records 67 did not contain sufficient data for the entire study period: In 65 cases the families had moved from one city to another within the first 3 years of life, and the new GP did not return information from the former. In two cases the records were handwritten and unreadable. The final population for this study was therefore 260 children. 227 children (87%) attended all the study visits, 25 (10%) missed one visit, 7 (3%) missed two visits and one child (0.3%) missed three visits.

For the 260 children a total of 6134 medical events were recorded in the COPSAC database. In 586 cases a medical event was registered by the GP but not by COPSAC.

Table 1 displays the completeness of the groups. The overall completeness including all the medical events was 0.91 [0.90;0.92)]. Completeness for the atopic diseases alone was 100%. Furthermore, there were no wheezy episodes registered by the GP that was not recorded in the diary or subsequently reported at the interview, indicating high awareness about project participation among both parents and doctors.

The completeness differed significantly (p < 0.0001) between the four remaining disease groups.

Table 2 displays the relationship between completeness and child’s asthma and socioeconomic status. The overall completeness was not associated with either child’s asthma (p=0.27), mother’s education (p=0.19) or mother’s occupation (p=0.28). The association with household income was borderline significant (p=0.06) with the completeness highest for the high income group (0.93 [0.91;0.94]) and lowest for the low income group (0.89 [0.86;0.91]) compared to the average income group (0.91 [0.90;0.93]). We also looked at the relationship between completeness within the five disease groups and asthma and socioeconomic status and found no significant associations.

**Table 2:** Completeness on overall illness in subgroups

	**Completeness**
**Child's asthma status**	*p = 0.2698*
With asthma (48)	0.91 [0.90,0.92]
Without asthma (212)	0.90 [0.87,0.92]
**Household income**	*p = 0.0589*
High (65)	0.93 [0.91,0.94]
Medium (125)	0.91 [0.90,0.93]
Low (69)	0.89 [0.86,0.91]
**Mother's education**	*p = 0.1937*
Elementary (103)	0.90 [0.87,0.91]
College (62)	0.92 [0.90,0.94]
Medium (64)	0.92 [0.90,0.94]
University (31)	0.93 [0.89,0.95]
**Mother's employment**	*p = 0.2842*
Professional (114)	0.92 [0.90,0.94]
Non-professional (96)	0.91 [0.89,0.92]
Unemployed (24)	0.88 [0.82,0.92]
Student (26)	0.91 [0.87,0.94]

Number of patients in each group in parenthesis, 95% Confidence intervals in brackets.

## Discussion

### Main findings

Doctor’s interviews at six-monthly clinic visits on child’s illness and symptoms supported by daily diary cards on lung symptoms provided 100% completeness to the main objectives of the study, the atopic diseases. Furthermore the current study revealed completeness above 90% to other respiratory and skin related illness and childhood infections. Other illnesses were only captured with a lower completeness around 77%. We saw an insignificant trend of better completeness in families from higher social status, but no influence from child’s asthma status.

### Strengths and limitations

The strength of our data also relates to the close longitudinal surveillance at the COPSAC clinical research unit. The study is a single-centre study with six-monthly assessments by experienced study-doctors examining and taking clinical history based on standard operating procedures supported by diary cards on atopy-related symptoms. This assures consistency in procedures, definitions of conditions and data capture methods and reduce risk of misclassification. Atopic disorders such as recurrent wheeze, asthma and atopic dermatitis in young children display significant between-observer variation [16-18]. The risk of misclassification is important particularly in young children with respiratory and skin disorders because there is gross inconsistency among doctors in their diagnostic and treatment practice, reflecting little consensus on definition and best practices.

It is a strength of our study is that we have provided an external reference to validate symptom history, but this choice can be debated. Previous reports have indicated that morbidity recorded by GP is a reliable estimate of community morbidity [19,20], and inter-observer differences have revealed satisfactory agreements between GPs around 80% [21,22]. Unfortunately GP records are only kept by the GP personally. So far there is no central registration of GP diagnose coding or link to a national database. The gate keeper function of the GP and the automatic notification of all health care contacts make the GP record a very reliable source of information on health care interactions. The fact that health care is free to all Danish citizens minimizes socioeconomic status as a significant confounder of contacts to the health care system.

Nevertheless, the study is limited by the fact that the GP record can only give information on illness where parents considered medical attention necessary. In fact, it is known that a large proportion of childhood illness resolves spontaneously and is not reported to the professional health care [23,24]. It is a further limitation that because of the nature of our data collection we only deal with the issue of under-reporting, i.e. sensitivity, but not over-reporting, i.e. specificity. We only captured missed diagnoses, but we cannot know the degree of over-reporting of illness, which was previously reported as a problem in interview surveys [3,25].

It is a limitation that our results relate only to interviews done six-monthly and do not permit us do draw conclusions on interview studies with shorter or longer intervals. We allowed a time span of 4 weeks between family practitioner and COPSAC registration of events. This choice is debatable since several events in either source could occur within this period and potentially lead to an underestimation of unreported events.

It is a limitation that we only received GP records with full follow up from 260 out of 350 children (74%). However, it can be argued that the individual GP’s willingness to provide COPSAC with sufficient data can be considered independent of the parent’s study compliance.

Socioeconomic status was determined at one year of age, but household income, educational and occupational status are dynamic and can change over the course of a study. However this study is carried out in a relatively short period of time, so we consider status at one year of age as a reasonable estimate for the entire study period. Another limitation in measuring socioeconomic status is that only education and occupation of the mother, but not the father was included. We used univariate analysis; this choice can also be questioned, since education and income are likely to be correlated and interactive effects can be missed by this procedure, although consensus on this matter is not clear [26].

The generalizability of our conclusions to a general population can be questioned because of the high-risk nature of the population. Subgroup analysis showed that the completeness of symptom history was independent of the child’s asthma symptoms, but the fact that all mothers had a history of asthma is likely to increase their awareness of symptoms associated with lung symptoms.

### Interpretation

This study primarily addresses the validity of COPSAC data. It also addresses the reliability of information on child health collected by parental interviews. The results can be beneficial to researchers working with cohort studies investigating pediatric public health, particularly atopic diseases. Data on illness are not perfect, but acceptable. The majority of studies on general illness in early childhood in Denmark are either retrospective [27], cross-sectional [28] or follow children for a shorter period of time [23,29], and little is known about the validity of such data. We would expect them to be less accurate without the close follow-up that the clinical cohort study provides, but further studies on this subject would be of great value.

Parents of children with many symptoms may be suspected to be more compliant with the project than the parents of children without symptoms, which could introduce confounding to further analysis. However, subgroup analysis showed sensitivity was independent on the child’s asthma.

Data on non-infectious events are not appropriate for further analysis due to the low completeness. Comparison of medical events with low sensitivities to those with higher suggests that understanding of the concept of illness influences reporting (Table 2). When asked about illness in childhood the parents preferentially report infectious diseases or diseases with similar symptoms, even though the interviews specifically invite any kind of illness. Participating in a study focused on asthma, eczema and allergy, parents may consider common events such as conjunctivitis and thrush as everyday complaints not severe enough to be considered as illness, and non-infectious events such as fractures, burns and wounds are not considered relevant for reporting.

We saw an insignificant trend of better completeness in families from higher social status. Although insignificant in this study, it may be prudent to consider this as a potential confounder.

## Conclusion

Clinical interviews of parents at six-monthly intervals shows complete sensitivity to the study objectives of child’s atopic disorders and very high sensitivity to other respiratory and skin disorders with no important influence from socioeconomic status or concurrent asthma. Our findings support the completeness of prospective doctor’s interviews at the clinic supported by diaries when assessing childhood health and symptoms related to atopic disease.

## Abbreviations

COPSAC: Copenhagen Prospective Studies on Asthma in Childhood; GEE: Generalized Estimating Equation; GP: General Practitioner; ICD10: International Classification of Diseases Version 10, WHO; ICS: Inhaled Corticosteroids; SAS: Statistical Analysis System.

## Competing interests

The authors declare that they have no competing interests.

## Authors’ contributions

NHV is responsible for the acquisition of data, obtaining and reviewing records from GPs, data analysis and interpretation, important intellectual input and writing of the manuscript. SMJ performed the statistical analyses and contributed with important intellectual input. HB is responsible for the integrity of the study as a whole, from conception and design to acquisition of data, analysis and interpretation of data and writing of the manuscript. All authors read and approved the final manuscript.

## Pre-publication history

The pre-publication history for this paper can be accessed here:

http://www.biomedcentral.com/1471-2288/12/160/prepub
